# Shedding light on night‐time erections: Determining the feasibility of nocturnal erection detection with penile transdermal light reflection of haemoglobin

**DOI:** 10.1002/bco2.410

**Published:** 2024-07-16

**Authors:** Evelien J. Trip, Hille J. Torenvlied, Henk W. Elzevier, Rob C. M. Pelger, Jack J. H. Beck

**Affiliations:** ^1^ Department of Urology Leiden University Medical Centre Leiden The Netherlands; ^2^ Department of Urology St. Antonius Hospital Utrecht The Netherlands; ^3^ Department of Medical Decision Making Leiden University Medical Centre Leiden The Netherlands

**Keywords:** erectile dysfunction, light reflection of haemoglobin, nocturnal erections, penile temperature, RigiScan

## Abstract

**Objectives:**

Utilizing penile saturation and temperature measurements presents a promising avenue for the development of an innovative sensor system aimed at nocturnal erection detection. This study aims to determine the feasibility of erection detection with light reflection of haemoglobin (LRH), as a precursor for penile saturation measurements, as well as penile temperature by comparison with simultaneous overnight RigiScan measurements.

**Materials and Methods:**

This is a proof‐of‐concept observational study on 10 healthy volunteers with a cross sectional design. A penile transdermal haemoglobin and temperature sensor was developed to measure penile LRH through real‐time monitoring with receiving photodiodes and emitting light‐emitting diode (LED). Besides statistical analysis on LRH, temperature and RigiScan data, a visual assessment was done to determine detectability of changes in the LRH and temperature course during the RigiScan‐annotated erections.

**Results:**

A total of 40 nocturnal erections from 10 healthy volunteers were annotated with the RigiScan. The LRH values significantly increase during a nocturnal erection (*p* < 0.01) and penile temperature (*p* < 0.01). The largest elevation of temperature was seen in the last erection, with an increase of 0.94°C. The corrected temperature shows an increase of 1.29°C in the last erection. Furthermore, visual detectability was feasible for 80% of the erections with LRH values and 90% with the temperature output.

**Conclusion:**

Penile LRH and temperature have the potential to serve as an alternative methodology for nocturnal erection detection compared with the currently applied circumference and rigidity measurements. This is an important step in the development of a patient‐friendly and modernized tool for erectile dysfunction diagnostics. An improved sensor should be developed to allow for calculation of saturation percentage from LRH values. In combination with penile temperature measurements, this allows for conduction of further validity studies to work towards translation into clinical practice for non‐invasive ED diagnostics.

## 1 INTRODUCTION

Erectile dysfunction (ED) is defined as the persistent or recurrent inability to attain or maintain an erection sufficient to perform sexual activity.^1^, ^2^ According to a study by Schouten et al., the incidence rate of ED is 19.2 per 1000 men annually, and it tends to be associated with higher age.^3^, ^4^ ED significantly impacts both physical and psychosocial well‐being.^4^, ^5^ Moreover, organic ED has emerged as a crucial sentinel marker of cardiovascular conditions.^6^, ^7^ Consequently, timely and accurate diagnosis of ED is essential for both adequate treatment of the dysfunction, as well as early identification of other potentially life‐threatening conditions.^8^

To diagnose the cause of ED, a non‐invasive diagnostic tool the RigiScan (GoTOP Medical, Minneapolis, MN, USA) was developed in 1985 by Bradley et al.^9^ The RigiScan measures nocturnal erections that occur during the rapid eye movement (REM) sleep. This gold standard tool detects the presence of nocturnal erections through penile circumference and rigidity measurements. It has been instrumental in diagnosing and understanding ED in men. Despite historical advantages, the RigiScan has many disadvantages in present time; the CE‐approval of the system is expired, the software is not compatible with modern devices and the device causes sleep disturbances resulting in questionable validity of test results.^1^, ^10^, ^11^

In recent years, there has been a growing interest in the use of sensor technology for the diagnosis of ED.^1^, ^11^, ^12^ In the era of wearable healthcare sensors and smartphone applications, an opportunity has arisen for the development of a ‘smart device’ tailored for optimized ambulatory nocturnal erection detection. A sensor can provide valuable information about the underlying causes of ED and can be used to tailor treatment to the individual patient.

RigiScan diagnostics is based on measurements of penile circumference and rigidity, which causes pressure on the penis resulting in discomfort. Recently, the study by Torenvlied et al. showed that penile temperature measurements can determine the presence of erections in healthy men.^13^ However, the results showed that duration and strength of erection cannot be determined by solely using temperature measurements. Thus, extending this system seems to be crucial.

While looking for a patient‐friendly alternative, the three important factors of normal erectile functioning can be considered: haemodynamic interaction of cavernosal arterial inflow, perfusion pressure and the development of an adequate degree of venous outflow resistance.^14^, ^15^ A full erection is accompanied by an absence of inflow and outflow of blood in the corpora cavernosa. The European Association of Urology guidelines state that patients with low flow priapism have blood saturation decline in the corpora cavernosa up to values below 30%.^16^ Blood saturation decreases are expected to also occur during normal erection. Edgar et al.^11^ suggest that because saturation can be measured using a small sensor system, this seems like a feasible alternative for nocturnal erection detection.^11^ Yet, no studies have investigated the feasibility of nocturnal erection detection with penile blood saturation measurements.

The primary aim of this study is to test the above‐mentioned hypothesis that nocturnal erections of healthy young men can be measured with light reflection of haemoglobin (LRH), which functions as a precursor system for penile saturation measurement. The secondary aim is to validate the outcomes of the study by Torenvlied et al.^13^ on penile temperature increase during nocturnal erection.

## 2 MATERIALS AND METHODS

### 2.1 Study setting and participants

This was an experimental observational study to test the above‐mentioned hypothesis. This study was conducted at the Urology department of the St. Antonius Hospital in Nieuwegein, the Netherlands. After approval of the Medical Research Ethics Committees United with study registration number R21.015 on 15 April 2021, informed consent forms from the volunteers were obtained between 20 June and 12 September 2022. The study was registered on Central Commission Human Research number: NL75891.100.21 and registered on ClinicalTrials.gov number: NCT05219071.

The findings of the ‘Feeling Hot’ study^13^ were followed to determine the sample size. In this pilot study, 10 healthy male volunteers between 18 and 40 years without (a history) of ED and willingness to sign informed consent were included. From the included test subjects, demographic data, including age, partner status, comorbidities, surgeries, medications and endothelial risk factors (diabetes, smoking, hypertension and hyperlipidaemia), were recorded. An IEFF‐5 (International Index of Erectile Function) questionnaire was taken, to ensure normal sexual functioning. Exclusion criteria were test subjects with sickle cell anaemia and volunteers with an IEFF‐5 below 22.

### 2.2 Study procedures

The LRH sensor was placed at the dorsal left side of the penis. This location was chosen due to the closest proximity to the corpora cavernosa without interference from the dorsal veins. The temperature sensor in the device was placed towards the top of the penis, as studies have shown that the temperature increase was highest proximally.^17^ The second sensor was placed on the left thigh, of which only the temperature measurement was used to calculate the corrected temperature (*T*_penis_ − *T*_thigh_). The sensors were placed by the researcher at the outpatient clinic. The volunteers placed the RigiScan before going to sleep. After the overnight measurements, the volunteers turned off and disconnected all sensors. If the RigiScan showed absence of nocturnal erections, the volunteers were asked to sleep for an extra night with all sensors, up to a maximum of three nights. Individuals who withdrew or who did not have nocturnal erections after three nights of measurements were substituted.

### 2.3 Sensor and data processing

The LRH sensor version 1.1 is a small portable device intended for measuring tissue oxygenation of the cavernous tissue of the penis in a flaccid and erect state. The MAX86141 Evaluation System is incorporated in a biocompatibility certified carbon fibre‐reinforced housing, measuring 39 × 17 × 10 mm, with a weight of 9 g.^18^, ^19^ The system contains a Bluetooth Low Energy (BLE) unit and a lithium‐ion polymer rechargeable battery. Furthermore, the sensor monitors a clock, calendar and data storage for over 20 h. The device is designed to investigate reflection of haemoglobin molecules in the penis through real‐time monitoring with two receiving photodiodes and one emitting light‐emitting diode (LED) with a wavelength of 536 nm. The two optical photodiodes measure reflected light and ambient light from the penis. The illuminating LED makes it possible to measure a difference in the LRH molecules. Although the transversion of light‐transmission values to SpO_2_ requires a second LED, it was chosen to first investigate the ability of detecting overnight penile LRH values during RigiScan‐annotated nocturnal erections. The novel penile sensor also includes a skin thermistor temperature sensor, based on the MAX30208 Evaluation System, which operates at low‐power with an accuracy of 0.1°C from +30°C to +50°C.

The measured LRH and temperature data are logged on a microcomputer. After the nocturnal measurement, the data are transferred through Bluetooth (low energy) to a computer for further data storage and processing in Microsoft Excel 365 version 2211.

The RigiScan was applied in this study as a gold standard to annotate the presence of erections. Sufficient nocturnal erections with the RigiScan were defined based on the criteria from Knoll and Abrams.^20^:

- erection duration ≥10 min;
- increase in penile tumescence ≥2.5 cm;
- penile rigidity ≥60%.^20^, ^21^

The RigiScan images were inserted with MATLAB (release R2019a), and through usage of the tool ‘ginput’, the start and the end of the erection were extracted with the predetermined criteria. From these startpoints and endpoints, the duration of the erection was calculated.

For each of the three parameters (LRH, penile temperature and corrected temperature), three outcome variables were calculated: first, a baseline value, defined as the minimum value recorded during the 15 min preceding the nocturnal erection as measured with the RigiScan; second, a peak value, which is the maximal value during the erectile phase; and finally, the delta value, defined as the difference between the peak minus the baseline value.

### 2.4 Statistical analysis

Descriptive and observational statistics were used to analyse data of the measurements of the RigiScan and the novel sensor. From all volunteers, the demographic and clinical characteristics, such as the average age and comorbidities, were determined through descriptive statistics given with a mean (SD), range or percentage (%).

Data were analysed with SPSS release 26 (SPSS Inc., Chicago, IL, USA). Significant difference in baseline and peak LRH, temperature and corrected temperature was tested with the use of a Wilcoxon signed rank test. Linear regression analysis was performed to test the relationship between erection duration and LRH increase and (corrected) temperature increase. Significance was stated at alpha <0.05 for the entire study.

A moving average filter with a sampling frequency of 100 samples was applied. Afterwards, all data were plotted, and figures were created for LRH values, penile temperature and corrected temperature to analyse visual detectability of the nocturnal erections and compare this with the data from the RigiScan.

For the visual assessment, the following criteria were stated for ‘visual detectability’: a peak in LRH or (corrected) temperature output that was higher for at least half of the duration of the erection measured with the RigiScan, compared with the observation period (15–60 min) preceding the RigiScan‐annotated erection.

## 3 RESULTS

Ten healthy male test subjects were included in the study. Average age of subjects was 29 years (range 21–39). Average BMI was 22.9 kg/m^2^ (SD 1.9). The IEFF‐5 average score was 24.2 (range 24–25). Eight subjects did not smoke including three former smokers. None of the test subjects had a history of ED, hypertension, hyperlipidaemia, sickle cell disease, atherosclerosis, sleep disorders, depression or apnoea. One subject had a well‐controlled diabetes mellitus (DM) type 1, wherefore insulin treatment. Another subject used antihistamine for allergies.

### 3.1 RigiScan

A total of 40 erections were seen in the RigiScan data, established according to the previously described criteria for RigiScan‐annotated erections. An average of four nocturnal erections per subject was measured (range 2–6). Average duration of an erection was 35 min (SD 23 min). Average diameter measured with the RigiScan in flaccid condition was 8.0 cm (SD 0.6) and in erectile condition 10.3 cm (SD 0.1).

### 3.2 LRH

A LRH increase was seen in all erections with an average increase from baseline 286 019 (SD 67 987) to a peak value of 347 512 (SD 103 457), resulting in an increase of 61 493 (SD 68 282) (see Table 1). The difference between baseline and peak LRH was significant (*p* < 0.01). Table 1 illustrates a smaller increase in LRH in the first and last erection, the largest difference in LRH was seen in all the erections measured.

**TABLE 1:** Overview of the measurement values for all erections and specified for first and last erection.

Variable	All erections (SD)	First erection (SD)	Last erection (SD)
Duration erection (in min)	35 (23)	30 (11)	38 (22)
LRH baseline (raw value)	286 019 (67 987)	278 743 (19 019)	277 771 (62 298)
LRH peak (raw value)	347 512 (103 457)	317 653 (22 655)	318 669 (88 768)
Difference in LRH (raw value)	*61 493 (68 282)*	38 910 (3636)	40 898 (30 575)
Temperature baseline (in °C)	34.54 (0.88)	34.72 (0.15)	34.47 (0.90)
Temperature peak (in °C)	35.33 (0.62)	35.35 (0.3)	35.40 (0.74)
Difference in temperature baseline (in °C)	0.79 (0.57)	0.62 (0.15)	*0.94 (0.45)*
Corrected Temperature baseline (in °C)	0.13 (0.82)	0.26 (0.92)	0.07 (1.00)
Corrected Temperature peak (in °C)	1.13 (0.82)	0.90 (0.58)	1.36 (1.09)
Difference in corrected temperature baseline (in °C)	1 (0.72)	0.64 (0.34)	*1.29 (1.03)*

*Note*: Values were given in mean (SD). Italics are used to highlight the biggest difference in the value.

**FIGURE 1:**
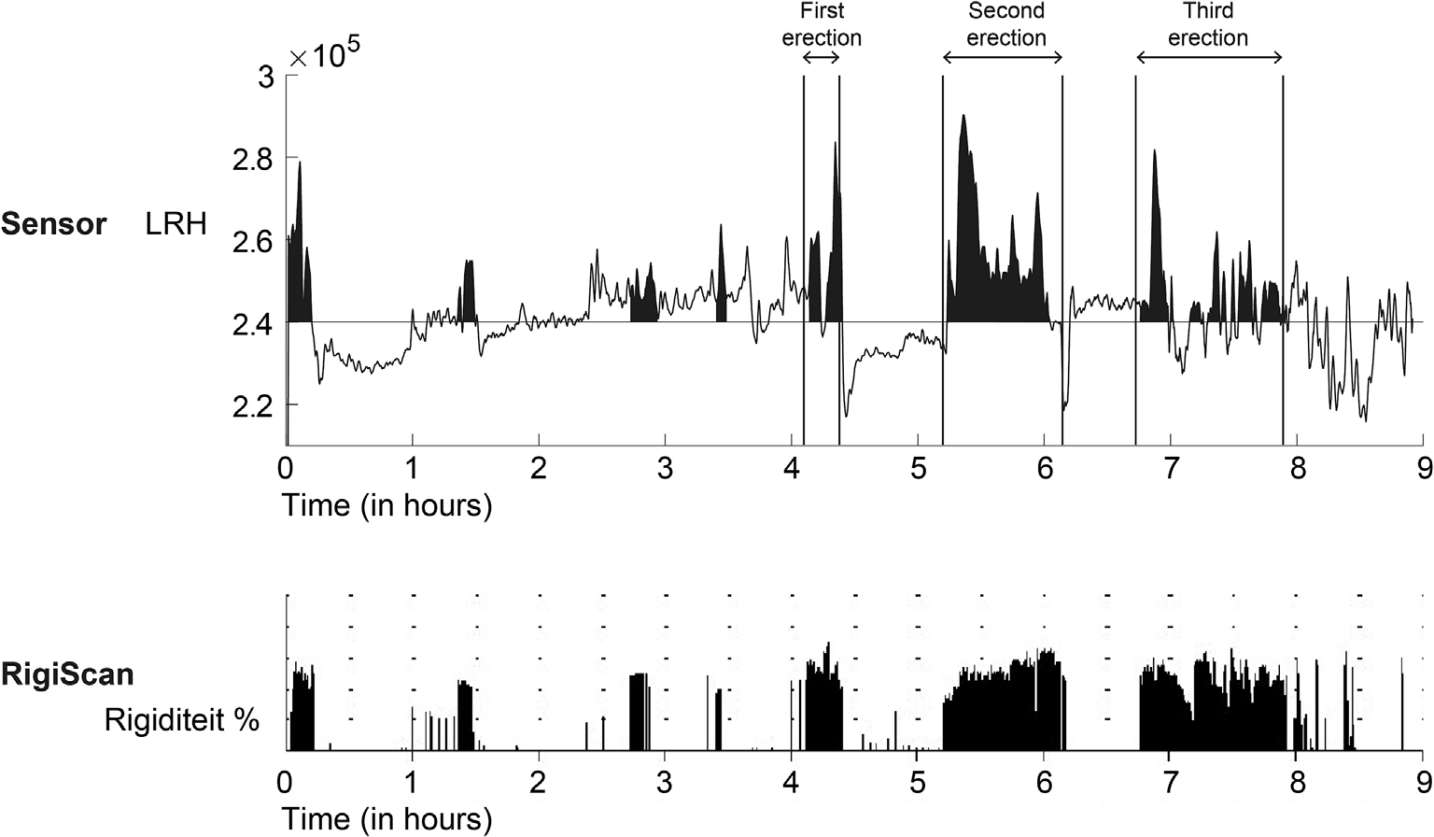
Outcome data of an overnight measurement of penile LRH and rigidity from the RigiScan of a single test subject.

Figure 1 showed the combined visual data of a nocturnal measurement of penile LRH and rigidity from the RigiScan. The first four rigidity peaks from the RigiScan were not included in the analysis, as they did not meet the previously mentioned requirements. However, in these rigidity peaks, an increase in LRH value was observed. The first full erection (starting at *t* = 4.1 h) and the second full erection (*t* = 5.2 h) detected with the rigidity of the RigiScan showed a LRH peak. In the time between these full erections, the LRH curve was stable. The third full erection (*t* = 6.8 h) showed peaks in the LRH followed by continued activity, which was also seen in the rigidity course of the RigiScan measurements.

**FIGURE 2:**
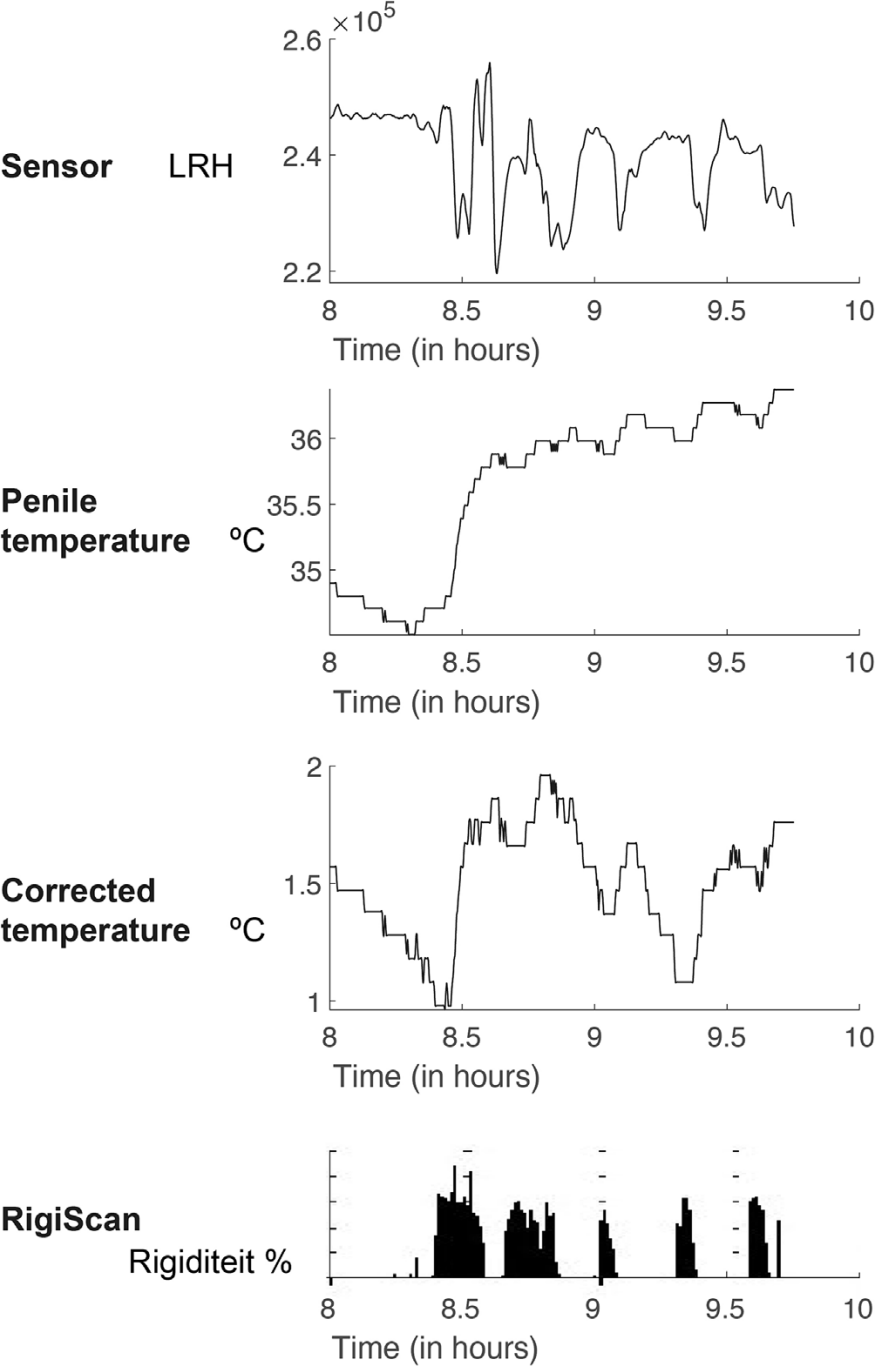
Outcome data from a different patient showing the last 2 h of the overnight measurement. The top graph shows the LRH value, below that the temperature, then the corrected temperature and below the values of the rigidity of the RigiScan. Here, you can see that even short erectile events (that were not included because of short duration or rigidity <60%) were visible with LRH and (corrected) temperature.

Figure 2 illustrates the occurrence of brief erections in a volunteer (at *t* = 8.45, 8.75, 9, 9.45 and 9.45 h); however, even these fleeting erections remained discernible when visualizing the LRH peaks. This effect was seen in the data of several volunteers.

After conducting the visual assessment of the curves of the LRH values measured by the novel penile sensor as described above, it was determined that 80% of the erections were detectable. Table 2 gives a detailed overview of the outcomes of the visual assessment.

**TABLE 2:** Visually detected erections with the novel sensor compared with the RigiScan.

Volunteer	1	2	3	4	5	6	7	8	9	10	Total
Number of erections with the RigiScan	6	4	3	3	3	2	5	4	6	4	40 (100%)
LRH peaks	6	3	3	3	3	1	4	2	4	3	32 (80%)
No LRH peak	0	1	0	0	0	1	1	2	2	1	8 (20%)
Temperature peaks	6	4	3	3	3	2	5	3	4	3	36 (90%)
No temperature peak	0	0	0	0	0	0	0	1	2	1	4 (10%)
Corrected temperature peaks	6	4	3	3	3	2	5	4	5	2	37 (93%)
No corrected temperature peak	0	0	0	0	0	0	0	0	1	2	3 (7%)

### 3.3 Penile temperatures

A temperature increase was seen in all erections averaging at 0.79°C (SD 0.57) with a baseline value of 34.54°C (SD 0.88) and an average peak value of 35.33°C (SD 0.62). The largest elevation of temperature was seen in the last erection, with an increase in the penile temperature of 0.94°C (SD 0.45) and a corrected penile temperature increase of 1.29°C (SD 1.03) (see Table 1). The penile temperature increase was significant (*p* < 0.01). Linear regression analysis showed a significant positive relation between temperature increase and the duration of the erection (*p* = 0.043).

**FIGURE 3:**
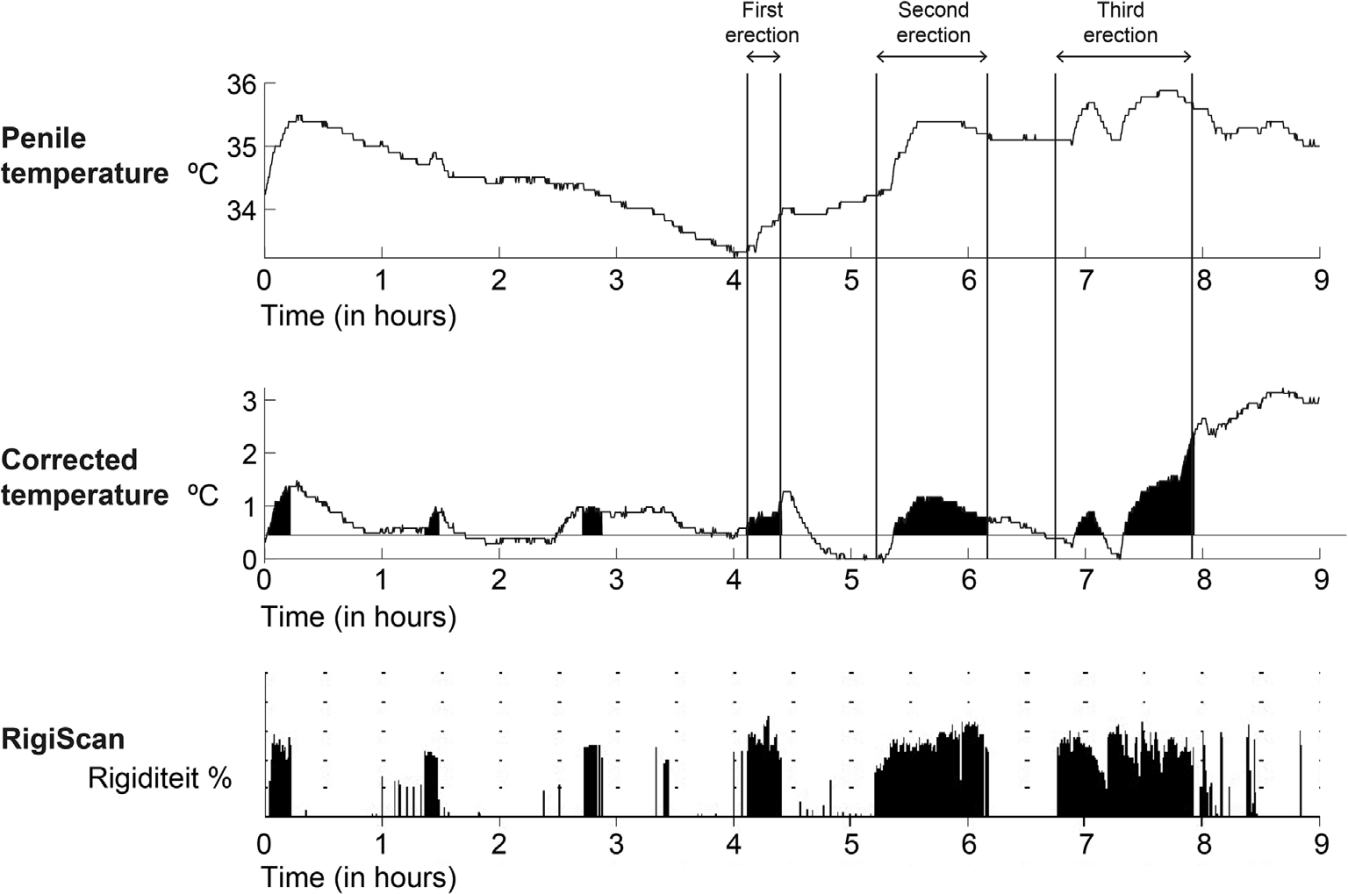
The outcome data from the penile temperature and corrected temperature measurements from one volunteer during an overnight measurement.

Figure 3 shows the overnight temperature data for one participant. The curve of the penile temperature was shown on top and the corrected temperature in the middle and the RigiScan rigidity data at the bottom. The first four rigidity peaks were not included in the study as full erections as they did not meet the previously mentioned requirements. It can be noted that a peak in (corrected) temperature was observed in the first two rigidity peaks. The first full erection (starting at *t* = 4.1 h) and the second full erection (*t* = 5.2 h) show a clear increase in both the penile and corrected temperature. The third full erection (*t* = 6.8 h) shows two peaks in the (corrected) temperature, which follows the rigidity course of the RigiScan measurements.

After analysing all the data following the previously described analysis protocol, a penile temperature peak could be detected in 90% of the erections annotated with the RigiScan. The corrected penile temperature peaks were visually detectable in 93% of the erections annotated with the RigiScan. Table 2 gives a detailed overview of the outcome of the visual assessment.

## 4 DISCUSSION

This is the first study to investigate the possibility of nocturnal erection detection in healthy volunteers with a wearable health sensor that measures both the LRH and temperature of the penis. The results of this study show a significant increase in penile LRH and penile temperature during nocturnal erections. Furthermore, the sensor allows for visual detectability of nocturnal erections in 80% to 93% of the erections annotated with the RigiScan.

This study is the continuation of the previous study of Trip et al.^22^ in which the LRH levels were measured in patients with ED after provoking an artificial erection with the use of alprostadil injected in the penis. The first study of Trip et al.^22^ was done with the LRH sensor version 0.1. In this study, similar LRH values and LRH differences (LRH baseline of 288 994 before an erection and 322 515 during erection) during erection were found. This supports the viability of utilizing LRH for the detection of erections in males.

The main limitation of this study is the application of LRH measurements instead of saturation outcomes. It is important to note that the measurement principle of the sensor applied in this study corresponds fully with that of a SpO_2_ sensor. However, it misses a second LED, which is required to translate LRH values into SpO_2_ percentages.

Besides distinguishing between psychogenic and somatic ED through measurement of SpO_2_ percentages, we hypothesize that it may be possible to distinguish between an arterial or venous cause of ED. It is expected that in arterial ED, there is a chronic lower saturation and a more constant temperature, following the results of the study by Brown et al.^23^ They found a low PO_2_ measured with blood gas after injection of vasoactive agent in patients with arteriogenic impotence. In patients with venous ED, it is hypothesized that there is a cavernosal saturation increase during REM sleep but no real saturation decrease as the blood is not retained in the corpora. This could be combined with a temperature increase due to the increased inflow of blood. Thus, an increase in temperature and oxygenation, without the concomitant decline in saturation, would be suspect for venous leakage. In the study of Padmanabhan and Mccullough,^24^ a local tissue StO_2_ measurement was placed on the penis before and after medication to induce an erection; men with ED had significantly lower corporal penile StO_2_ levels compared with men without ED. There was a significant improvement of StO_2_ levels in ED patients after using medication to induce an erection. Unfortunately, there was no mentioning about the state of the penis (flaccid or erect).

Analysing the data, the sensor sometimes did not make contact with the skin during an erection. We hypothesize that this is the result of differences in increase in circumference of the penis during an erection. The sensor cover was curved based on an average penile circumference of 9.39 cm.^25^ An updated version of the sensor should have multiple available covers for different penile diameters or a semi‐flexible case that bends following the change in penile circumference.

The outcomes of the temperature measurement of the penis in the current study are in line with the outcomes of a study by Torenvlied et al.^13^ This study investigated the feasibility of nocturnal erection detection with the penile temperature methodology in a similar population and study set‐up.^13^ That study showed an average penile temperature increase of 0.87°C during nocturnal erections compared with 0.79°C in this study. In the current study, the largest temperature variance was seen during the last nocturnal erection (temperature increase of 0.94°C). In the study by Torenvlied et al.,^13^ the largest difference was seen at the initial erection with an increase of 1.47°C. This difference can be attributed to the current studys methodology, where the first erection occuring within 30 min of placing and activating the RigiScan was excluded, as it was not considered a nocturnal erection. In eight volunteers, some erectile activity was observed with the RigiScan within 30 min of placing the device. However, these particular erections were excluded in our study as they did not fulfil the criteria stated for erection annotation by the RigiScan. This phenomenon, known as a responsive erection, is unlikely to occur in patients with ED.

The positive correlation in penile temperature that was observed indicates that penile temperature measurements could also indicate erection duration to some extent, which was hypothesized as impossible in the ‘Feeling Hot’ study by Torenvlied et al.^13^ The ‘Staying Hot’ study^26^ revealed the influence of blankets and underwear on penile temperature during an erection, which caused penile temperature to remain elevated at peak temperature levels under blankets after an erection. Our study was conducted during summertime in the Netherlands. A large proportion of the volunteers slept without a blanket or under a thin sheet. This can explain the larger temperature variance in the last erection (with the longest duration), as the temperature could return to baseline levels as a result of increased heat conduction and enabled heat convection with decreased insulation of blankets. The outcomes of this study show the necessity of standardizing the overnight conditions during overnight temperature measurements for application of the temperature methodology. The approach should regard the effect on the sleep quality in the measurement set‐up.

Entering the bed also contributes to an increase in bodily and penile temperature; therefore, usage of only a penile temperature sensor is insufficient for measuring nocturnal erections.^26^ Adding the thigh sensor makes it possible to distinguish between temperature increase from absent convection and low conductivity of blankets or from a (responsive) erection. The corrected temperature, incorporating data from both sensors, exhibited the highest positive predictive value (93%) for nocturnal erection detection, emphasizing its essential role in the application for erection detection.

The interpretation of novel sensor data without the support of the RigiScan makes it demanding to detect all types of erections. The LRH graph (depicted in Figure 1) offers clearer visibility when an erection persists for an extended period. However, even the brief and less rigid erections were discernible in some volunteers through the novel sensor (Figure 2), posing a challenge in establishing a reliable negative predictive value for the novel sensor. In this study, the focus was on nocturnal erections, leading to the exclusion of short and/or less rigid erections.

## 5 RECOMMENDATIONS

This study successfully fulfilled the objective to investigate if the combined LRH and temperature sensor is able to detect nocturnal erections in a healthy male. The next step is to develop a saturation and temperature sensor and to validate this sensor in ED patients. Regarding the limitations of necessity to apply the RigiScan to annotate the nocturnal erections in the validity studies, it might be easier to compare the new sensor to an activity tracker (which, among other things, monitors sleep), because this can detect REM sleep. This could make the RigiScan redundant. The study of Liu et al.^27^ shows a potential link between sleep‐related erections and REM sleep measured with an activity tracker. Eventually, all this progress will make nocturnal home monitoring easier to perform. The RigiScan provides information about the quality, quantity and duration of the erection. The future sensor should be tested in different age groups with normal erections and in men with various causes of ED to ultimately make a quality indication as with the RigiScan and making it easier to use in clinical practice.

## 6 CONCLUSION

The significant increase observed in penile LRH and temperature during nocturnal erections marks an initial step towards developing a modernized, patient‐friendly tool for ambulatory ED diagnostics. Successful reproduction of over 80% of erections annotated with the RigiScan was achieved. Future research should focus on the feasibility of combining saturation percentage measurements with penile temperature assessments for easier ED diagnostics.

## AUTHOR CONTRIBUTIONS

Evelien J. Trip was responsible for conceptualization, methodology, formal analysis, investigation, data curation, writing and project administration. Hille J. Torenvlied contributed by doing data curation, reviewing and editing. Henk W. Elzevier, Rob C. M. Pelger and Jack J. H. Beck contributed with conceptualization, supervision and giving feedback on the report.

## CONFLICT OF INTEREST STATEMENT

The authors declare that they have no conflict of interest or competing financial interest.
